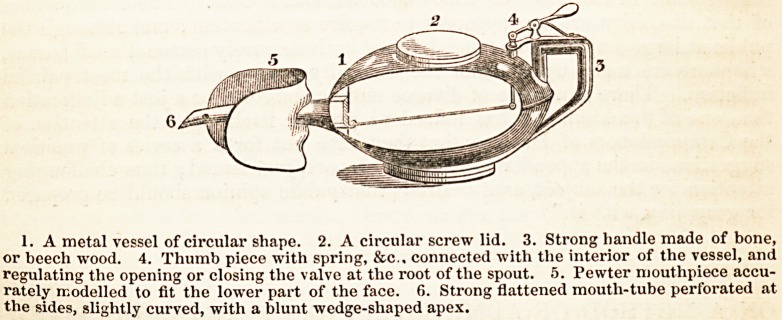# On a Method of Administering, by Means of a New Contrivance, Nourishment to Insane Persons Who Refuse Food

**Published:** 1853-04-01

**Authors:** John Foster Reeve


### ON A METHOD OF ADMINISTERING, BY MEANS OF A NEW CONTRIVANCE, NOURISHMENT TO INSANE PERSONS WHO REFUSE FOOD,

INVENTED BY JOHN FOSTER REEVE, M.R.C.S.E.

Few asylums fail to number, amongst the unfortunates confided to their protection, certain individuals whose particular delusion induces them to decline taking the slightest sustenance, and who persist in their refusal during days, weeks, or even months.* Stratagem and persuasion are alike unavailing to alter their determination, and various and singular are the causes assigned for their conduct by these poor victims of a terrible malady. An aged and emaciated woman, under the writer's care, declined eating from a dread of growing fat; and the case of a lady was recently presented to his notice, who

* It might be added, years; for Dr. Brown, of the Crichton Institution, Dumfries, mentioned in his Twelfth Annual Report, for 1851, the case of a female patient, then under his care, who had refused food for two years and one month.

obstinately refused food, seriously assuring those around her that we were only commanded to break bread, but to eat it was a very sinful act. Cases in which temporary loss of appetite arises from bodily indisposition, belong to another category, and need soothing measures; but when a suicidal patient meets his medical attendant with entreaties to be left undisturbed, as food is useless, and he desires to die; or others affirm that poison is mingled in their provision, and gravely state motives to justify abstinence, there remains no alternative but to administer by force sufficient nourishment for the support of the patient's existence, till his delusion disappears, or it changes its character.

Numerous methods have been devised for the forcible introduction of food, under such circumstances, into the stomach. The spouting boat, designated by Dr. Haslam, a "devilish engine;" the equally formidable instrument of his own construction; tubes of various shapes, have in turn found employment; success depending chiefly upon the operator's skill. At present, the stomach-pump is in more general use—a use demanding the greatest care, always attended with peril, and sometimes followed by even fatal effects. Amongst many accidents of actual occurrence, are recorded, injection of liquids into the lungs intended for the stomach; lacerating the walls of æsophagus; and tearing the mucous membrane into strips; blood vomited from ruptured vessels; and death, through suffocation, have also immediately succeeded the pumping operation.

The accompanying sketch represents an instrument designed and used by myself some years since, which has proved, upon repeated tests, perfectly adapted to its object, and can be employed without danger, difficulty, or inconvenience.

1. A metal vessel of circular shape. 2. A circular screw lid. 3. Strong handle made of bone, or beech wood. 4. Thumb piece with spring, &c., connected with the interior of the vessel, and regulating the opening or closing the valve at the root of the spout. 5. Pewter mouthpiece accurately modelled to fit the lower part of the face. 6. Strong flattened mouth-tube perforated at the sides, slightly curved, with a blunt wedge-shaped apex.

The patient placed recumbent upon a bed, his head resting on the pillow, and, as well as his body and extremities, firmly held by the assistants to prevent his moving, the spout of the vessel should be applied to his lips and a small quantity of the contents allowed to flow over them. The operator, holding and directing the instrument with his right hand, should with the thumb and finger of the left compress the patient's nostrils, thus compelling him to open his mouth in the involuntary effort to draw breath. The spout should then be quickly insinuated between his teeth, and the mouth-piece fitting to the face maintains the vessel in its position. The operator can regulate the flow of liquid by simply pressing his thumb upon the key at the top of the handle, while he observes the acts of deglutition, by watching the ascent of the larynx.

The writer subjoins an outline of several cases from the number that came under his notice, in which, by the simple method described, life was undoubtedly preserved, time obtained, and remedial measures rendered possible.

Case 1.—A. T., a female of healthy appearance, aged 38. She was married and the mother of several children; all her front teeth had been broken in former violent endeavours to administer food. She moaned incessantly, declaring she was the greatest sinner that had ever lived, and when offered nourishment, earnestly replied, "she was a dead woman, and how could the dead eat?" It was vain to remind her the dead also ceased to speak. Only with the utmost difficulty could she be made to endure any clothing; and, tormented by perpetual restlessness, seemed night and day incapable of sleep. Lord Byron pourtrayed the features of a similar condition in colours vivid and exact when he so beautifully described the madness of Haidee—

> "Food she refused, and raiment; no pretence
> Availed for either; neither change of place,
> Nor time, nor skill, nor remedy, could give her
> Senses to sleep—the power seem'd gone for ever."

In this instance, for a period of two months and fifteen days, the patient obstinately refused the slightest particle of sustenance. Liquid food, consisting of strong beef-tea, milk and arrowroot, was administered through the instrument three times a day, in quantities varying from half-a-pint and upwards, until with improved mental health her delusion gradually disappeared.

Case 2.—E. W., aged 24. A woman of delicate habit, the wife of a tradesman, whose infidelity is said to have induced the desponding state of mind that characterized her mental disorder. She imagined "her soul had gone out of her," and always walked in a stooping posture, because she affirmed her legs were not strong enough to bear the insupportable weight of her body. The poor creature became sullen, avoided conversation, and resolutely refused food. She was fed three times a day during ten days, when a favourable change ensued, and artificial means were no longer needed.

Case 3.—E. A., aged 60. A sturdy Irish woman, married, and the mother of two children; at the time alluded to she was the subject of a second attack of insanity, and a few days prior to admission into the asylum attempted self-destruction with a table-knife; failing in her object, she endeavoured to starve herself, and it became necessary to use the feeder for fourteen days, when the patient ceased to exhibit a suicidal impulse, and partook of food in the usual manner.

Case 4.—W. R., aged 44. Married, and the father of several children; he was suffering from a recent attack of acute mania, and extremely violent; although reduced to an emaciated condition by long fasting, he talked continually and incoherently upon religious subjects, and considered rigid fasting an act of piety; it was requisite to employ the feeder but for a few days only; the time gained and strength imparted produced however a most beneficial effect, and the patient eventually recovered.

Case 5.—W. S., aged 38. Married, and the father of two children. A strong, muscular man, of excessively irritable temper, and violent disposition. He had been for some years insane, and victim of various hallucinations. He styled himself the "morning star," and "the only son of the globe;" and fancied he possessed divine power. Occasionally, under the influence of violent paroxysms of excitement, he would refuse food. And, after allowing him a few days to re-consider the matter, the feeder was put in requisition. He did not like the process, and seldom obliged a repetition.

Case 6.—E. D., aged 25. Unmarried, and formerly a sempstress. A diminutive, deformed, and emaciated woman, with lateral curvature of the spine. Her mental disease had continued for above a year; and she advanced from occasional rejection, to the resolute refusal of food; stating that "she had made a vow not to eat." She would drink water, if she had seen it drawn, and felt assured no other nutriment was mingled in the cup. For two months and three days life was sustained by the artificial administration of nourishment.

It is unnecessary to multiply examples, and it would seem unnecessary to insist upon the value of time gained in similar cases, both for the preservation of the patient's life, and the chances of his ultimate recovery. But a suggestion has been made, that the idea of refusing food, conspicuous under some forms of insanity, is merely an instinctive sign to show the patient does not require it; his natural functions being in a temporary state of inaction or suspension; a theory thus opposed to the known laws of physiology falls inevitably before the test of practical observation. Two modes are left to pursue when an insane person persists in abstinence; either he must be suffered to perish from inanition, or food must be administered notwithstanding his resistance. The writer devoted considerable attention to the design of the simple instrument which he now recommends for the purpose, its extensive application would supply a want of suitable means he has himself frequently experienced in the course of his professional duties; a glance at the brief detail of instances cited will manifest that no circumstance arising from age, sex, or debility of constitution, nor from the violence of the patient, his strength, and determined resistance, nor the necessity of repetition through a lengthened period of the same forcible means, can interfere with the successful and perfectly safe operation of the instrument employed by the medical attendant, with the most ordinary degree of care.

---

## Figures and Tables

**Figure f1:**